# Add Bilingualism to the Mix: L2 Proficiency Modulates the Effect of Cognitive Reserve Proxies on Executive Performance in Healthy Aging

**DOI:** 10.3389/fpsyg.2022.780261

**Published:** 2022-01-31

**Authors:** Federico Gallo, Joanna Kubiak, Andriy Myachykov

**Affiliations:** ^1^Centre for Cognition and Decision Making, Higher School of Economics, Institute for Cognitive Neuroscience, Moscow, Russia; ^2^Centre for Neurolinguistics and Psycholinguistics, Vita-Salute San Raffaele University, Milan, Italy; ^3^Department of Psychology, Northumbria University, Newcastle upon Tyne, United Kingdom

**Keywords:** bilingualism, cognitive aging, cognitive reserve, cognitive reserve proxies, executive functions

## Abstract

We investigated the contribution of bilingual experience to the development of cognitive reserve (CR) when compared with other, traditionally more researched, CR proxies, in a sample of cognitively healthy senior (60 +) bilingual speakers. Participants performed in an online study where, in addition to a wide inventory of factors known to promote CR, we assessed several factors related to their second language (L2) use. In addition, participants’ inhibitory executive control was measured via the Flanker Task. We used Structural Equation Modeling to derive a latent composite measure of CR informed by traditional CR proxies (i.e., occupational complexity, marital status, current and retrospective socio-economic status, physical exercise, perceived positive support, maximal educational attainment, frequency of leisure activities and extent of social network). We examined whether bilingualism may act as a mediator of the effects of such proxies on cognitive performance therefore assessing the unique contribution of dual language use to CR. First, our analyses revealed facilitatory effects of both L2 age of acquisition and L2 proficiency on the executive performance. Second, our analyses confirmed the moderating role of bilingual experience on the relationship between other factors known to promote CR and cognitive integrity, revealing a strong contribution by bilingualism to CR development. Our findings provide further support to the notion that bilingualism plays an important role in mitigating cognitive decline and promoting successful aging.

## Introduction

A key concept when discussing prevention or mitigation of cognitive aging is that of cognitive reserve (CR; Stern et al., 2020). CR is defined as the discrepancy between the expected and observed levels of cognitive impairment, given the observed level of age-related neuropathology or brain disruption (Stern et al., 2020). In other words, CR is the individual ability to compensate for age-related neural deterioration and maintain optimal cognitive functioning. Many lifestyle factors have been suggested to promote successful aging by means of CR development (for a review see Cheng, 2016), and bilingualism has been implicated as one such factor (for a review see Gallo et al., 2020).

While evidence supporting the role of bilingualism as a CR promoter continues to accumulate (e.g., Gold et al., 2013a; Perani et al., 2017; Del Maschio et al., 2018), research on bilingualism-related benefits on cognitive aging seems to fail, up to this point, to dialogue with the research on more generally oriented CR factors. Importantly, bilingualism might play a unique role among the known CR proxies due to our knowledge of the route through which it affects aging, namely its putative beneficial role on executive functioning. Indeed, parallel activation of co-present linguistic systems has been extensively observed in the bilingual brain (e.g., Kroll et al., 2014). This simultaneous activation of competing information leads to a conflict, which must be successfully and rapidly resolved by the bilingual speaker. The cognitively effortful simultaneous management of two competing linguistic systems is governed by a *language control* device wired in a neural network that overlaps with the domain-general executive system (Abutalebi and Green, 2016). Thus, via this sustained control effort, bilingualism may act as a “cognitive gym” allowing daily training and strengthening of the executive control, both at the neural and the behavioral levels (Abutalebi and Green, 2016; Bialystok, 2017). This hypothesis is in line with training effects widely observed for other executive sub-systems, such as attention (Posner and Fan, 2008; Tang and Posner, 2009; Posner et al., 2015). Indeed, several investigations report that bilinguals outperform monolinguals in various executive functions tasks (for a review see Bialystok, 2017) and show increased structural density and functional efficiency in the executive network, relative to monolinguals (for a review see Li et al., 2014), across the lifespan. Given the fundamental role that executive functions play in the cognitive aging process (for a review see Reuter-Lorenz et al., 2021), the continuous executive training provided by bilingualism would end up supporting successful aging. Nonetheless, some degree of variability emerges in the literature when attempting to replicate such bilingualism-induced beneficial effects (see e.g., Paap et al., 2015). Such variability has been recently ascribed to the tendency of dichotomizing the spectrum of bilingual experience, i.e., favoring group comparisons between “bilinguals” and “monolinguals” over a detailed, continuous assessment of the individual bilingual experience (e.g., Luk and Bialystok, 2013; Surrain and Luk, 2019). To overcome this issue, in line with recent investigations (e.g., Hervais-Adelman et al., 2018; DeLuca et al., 2019; Gallo et al., 2021), here we operationalized bilingual experience continuously, for the first time to our knowledge in an investigation on senior individuals.

The abovementioned peculiar stance of bilingualism among CR-inducing factors, i.e., the extra insight into the cognitive domain acting as a relay for age-related beneficial effects, provides the ground to test bilingualism’s role as a CR factor in a novel way, namely assessing the extent of bilingualism’s contribution compared to those of other, traditionally more researched, CR proxies. To this end, here we investigated whether bilingualism may modulate the relationship between widely known CR proxies, namely physical exercise, education, occupational complexity, social network, and leisure activities (for a review see Cheng, 2016), and cognitive performance during senescence. Given the amount of available evidence on positive effects of bilingualism on executive functioning, a set of cognitive ability that is well-known to play a central part in the cognitive aging process (for a review see Luszcz and Lane, 2008), we expected to observe a modulation of such relationship, indicating a primary role of multiple language use in supporting successful aging and preventing age-related cognitive decline.

## Materials and Methods

### Participants

64 healthy older adults (30 males; mean age = 64.7, SD ± 4.7) were recruited via social media platforms (e.g., Facebook) and through the research recruitment platform Prolific^1^. The Psytoolkit software was used for data collection (Stoet, 2010, 2016). Requirements to participate in the study included at-least-partial knowledge of a second language (L2) and being aged 60 or above. Participants were screened for the presence of psychiatric or neurological impairments and those with a history of such impairments were removed from the analysis, resulting in the exclusion of one subject who reported an active Major Depression diagnosis. Participants also underwent an adapted online version of the Mini-Mental State Examination (MMSE; Cockrell and Folstein, 2002), to further control for the possible presence of age-related cognitive impairment in the sample. No participants were removed due to insufficient MMSE performance. Of the 63 participants eligible for the study, 36 spoke various first languages (L1s) and English as an L2, while the other 27 spoke English as an L1 and various L2s. We included the 36 L2 English speakers in the core analyses, while the full sample was used for sensitivity analyses and derivation of CR index (see in detail below). Participants were informed that they could withdraw at any point of the study and that all of the provided data would remain anonymous. Further, each subject was warned about potentially sensitive questions. On questions deemed as potentially upsetting, the option “I don’t know” was provided in order to allow participants to avoid answering. All participants provided informed consent to take part in the study.

### Demographics, General Intelligence, and Language Profile Assessment

All participants underwent a comprehensive online questionnaire in the Qualtrics platform that investigated their profiles in the following dimensions:

• Socio-demographics: age, sex, ethnicity, nationality, marital status, highest educational attainment;
• Physical health: nutritional status and dietary habits, cardiovascular health, neurological health, psychological health, presence of diabetes;
• Occupation: current and retrospective employment status, type of longest occupation, satisfaction with professional life;
• Retrospective socioeconomic status: parents’ occupation, presence of financial problems during upbringing;
• Social network: contact frequency with relatives, friends and neighbors;
• Leisure activities: participation frequency in different leisure activities;
• Physical exercise: frequency of low- and high-impact physical activities;
• Perceived positive support: level of satisfaction with the support received from contacts in (eventual) situations of need.

A separate section of the questionnaire investigated participants’ language background, including questions regarding L2 exposure, L2 proficiency, and number of years passed since L2 acquisition (henceforth, L2 years, a reversal of the age of acquisition measure devised to produce effects in the same direction of L2 proficiency and L2 exposure), as well as number of languages spoken. The full questionnaire can be found in Supplementary Material. Participants also underwent a subset of the Raven’s Standard Progressive Matrices for adults (Court and Raven, 1992) to assess their general intelligence, as well as the online Cambridge test for adult learners^2^ to assess their proficiency in English.

### Assessment of Executive Performance

To investigate participants’ executive ability, we presented them with a Flanker Task (Fan et al., 2005), a task measuring inhibitory executive control. In this task, a fixation cross is presented at the center of the screen for 400 ms, followed by an array of five arrows pointing to the left or to the right for a maximum duration of 2,000 ms. Participants are required to indicate the direction of the central target arrow by pressing the corresponding arrow key on the PC keyboard as accurately and fast as possible. Targets appear surrounded by flankers pointing to the same direction (→→→→→) (i.e., congruent condition), to the opposite direction (←←←←←) (i.e., incongruent condition), or by neutral dashes (– – → – –) (i.e., neutral condition). While congruent trials facilitate target response, incongruent trials present conflicting visual information and thus require inhibitory executive control to suppress its impact on the target response execution, typically entailing lower accuracy and longer reaction times (RTs). The three trial types were presented in a pseudo-randomized order during two runs of 96 trials each (32 for each condition). Participants were familiarized with the task via a practice run of 24 pseudo-randomized trials. The rationale behind the choice of this task lies in the fact that it mimics closely instances of bilingual language control, by relying on cognitive mechanisms such as conflict monitoring, interference inhibition and response selection, which are routinely required from bilinguals to carry out successful communication (Green and Abutalebi, 2013). For this reason, the Flanker task (sometimes replaced by the analysis of the sole executive component of the ANT task; Fan et al., 2005) is typically used in research on the cognitive consequences of bilingualism (Luk et al., 2010; Abutalebi et al., 2012; Del Maschio et al., 2018). As an additional reason behind our decision, we aimed to obtain results comparable to those of our previous investigations on the relationship between bilingualism and cognitive reserve, which all deployed the Flanker task (e.g., Del Maschio et al., 2018; Gallo et al., 2021).

### Statistical Analyses

**FIGURE 1:**
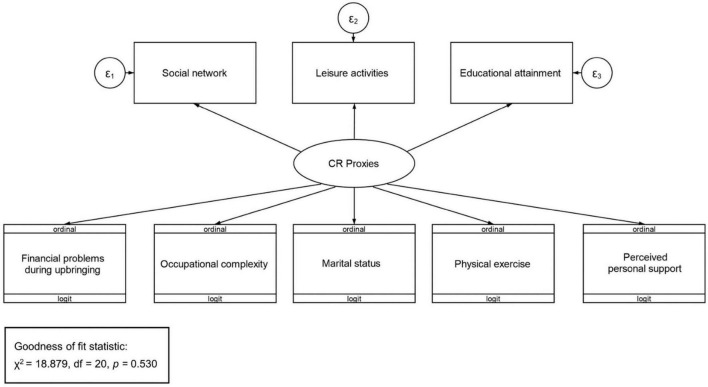
Structure of the structural equation model (SEM) used to derive the CR Proxies latent variable.

We used generalized Structural Equation Modeling (gSEM) in STATA 17 (StataCorp, 2021) to derive a latent measure of CR combining the contribution of different traditional CR proxy variables to a latent CR variable. The model (see Figure 1) included contributions from occupational complexity, marital status, presence of financial difficulties during upbringing, physical exercise and perceived positive support, as well as educational attainment, frequency of leisure activities and extent of social network. Since the first five predictors were categorical, an ordinal logit family link was used. For the last three, continuous, predictors, a linear family link was used. The corresponding linear SEM (STATA 17 does not allow postestimation of goodness of fit indices in the generalized SEM framework with mixed continuous and categorical predictors) fit the data well (χ^2^ of fitted vs. saturated model test = 18.879, df = 20, *p* = 0.530). Next, we predicted individual values of the CR latent variable, which was normally distributed with a mean of 0.

As per the Flanker data, we removed incorrect trials and false starts (i.e., RT < 100 ms), as well as outlier trials, namely trials with RTs falling beyond 3 SDs from the mean RT of each participant. Neutral trials were also discarded, since we focused on the conflict effect, a measure of inhibitory executive control calculated as the difference between RTs of congruent and incongruent trials.

#### The Impact of Bilingual Experience on Age-Related Cognitive Decline

**FIGURE 2:**
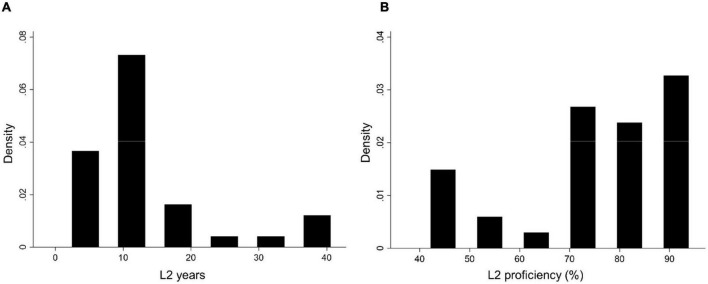
Histograms of the distribution of **(A)** number of years passed since L2 was acquired (L2 years) and **(B)** L2 proficiency in the L2-English subsample.

We aimed to investigate whether bilingual experience, measured as a continuous variable on three dimensions, namely L2 proficiency, L2 exposure and L2 years, impacts executive performance in healthy aging. Nonetheless, data on L2 exposure presented too little variability, and thus had to be excluded from the analyses: 80% of the participants reported to speak their L2 on a daily basis, while the remaining 20% was distributed across the four categories of weekly, monthly, quarterly or more rarely (see Figure 2 for the distributions of L2 proficiency and L2 years). To test our hypothesis, we used a by-trial linear mixed-effects approach, which made it impossible to compute the conflict effect in the traditional way, namely as the difference between average RTs in the congruent and incongruent conditions. Hence, since we were interested in the differential effect of bilingualism on incongruent trials, i.e., those tapping on executive inhibitory control, we inserted an interaction term by trial type for each of our predictors of interest. Our model thus included Flanker RTs as the dependent variable, L2 proficiency, L2 years (both in interaction with trial type) and trial type as predictors, age, sex and general intelligence as covariates, as well as random intercepts for participants and random slopes for trials.

#### The Impact of Bilingualism on the Relationship Between Cognitive Reserve Proxies and Cognitive Performance

Beside its consequences for cognitive performance *per se*, we aimed at testing whether bilingualism still exerted a beneficial effect on the cognitive aging trajectory when traditional CR proxies were also taken into account. Thus, we investigated whether L2 proficiency and L2 years modulate the well-known relationship between CR proxies and cognitive performance during senescence. To this end, we used a linear mixed-effects model including Flanker RTs as the dependent variable, three-way interactions between L2 proficiency, trial type and the CR proxies latent variable (henceforth, CR proxies) and L2 years, trial type and CR proxies, respectively, as predictors, age, sex and general intelligence as covariates, as well as random intercepts for participants and random slopes for trials.

#### Sensitivity Analyses

We additionally replicated the same set of statistical analyses in the whole sample of 63 individuals. The reason for this choice was two-fold: on the one hand, it allowed us to almost double our sample size, increasing statistical power; on the other, it allowed us to test whether the effects found for a sample of individuals sharing their L2 but differing in their native language also extend to individuals with a reverse linguistic profile, i.e., sharing their L1 but speaking different L2s. There was no significant difference in L2 proficiency or L2 years between the two subsamples.

## Results

### The Impact of Bilingual Experience on Age-Related Cognitive Decline

**FIGURE 3:**
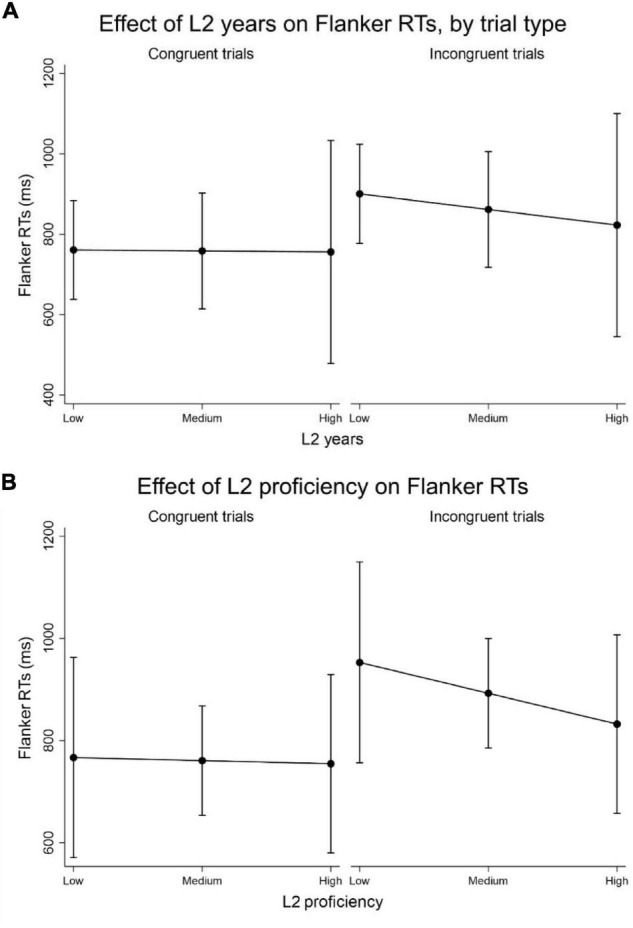
**(A)** Interaction plot for the L2 years * trial type interaction predicting Flanker RTs (in ms). Increasing levels of time passed since acquiring L2 predict lower RTs, i.e., better inhibitory executive performance; **(B)** interaction plot for the L2 proficiency * trial type interaction predicting Flanker RTs (in ms). Increasing levels of L2 proficiency predict lower RTs, i.e., better inhibitory executive performance.

The analysis revealed a significant effect of both L2 years (β = −2.797; *p* < 0.001) and L2 proficiency (β = −9.045; *p* < 0.001) on the executive performance of senior individuals, which differentially impacted congruent and incongruent trials. In particular, both variables beneficially affected the performance in the incongruent trials, but had no effect on the congruent trials, in line with the hypothesis that bilingualism enhances executive control abilities (see Figure 3). The beneficial impact of L2 proficiency was higher relative to that of L2 years.

### The Impact of Bilingualism on the Relationship Between Cognitive Reserve Proxies and Cognitive Performance

**FIGURE 4:**
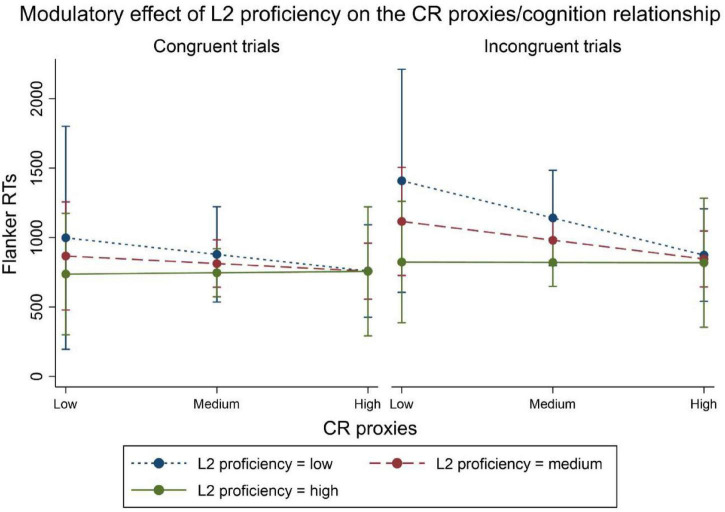
Interaction plot for the L2proficiency*trial type*CR proxies interaction predicting Flanker RTs (in ms). Higher L2 proficiency entails lower RTs, i.e., better inhibitory executive performance. Increases in the CR proxies score predict lower incongruent RTs only at low levels of L2 proficiency. At increasing levels of L2 proficiency, variations of CR Proxies score do not affect executive performance.

We registered a significant three-way interaction between L2 proficiency, trial type, and CR proxies (β = 45.276; *p* < 0.001), while it only approached significance for L2 years (β = 7.483; *p* = 0.092), consistently with the previous analysis showing a stronger contribution by L2 proficiency. The interaction plot (see Figure 4) revealed that in the incongruent trials, for increasing levels of L2 proficiency: (i) executive performance levels increased, irrespectively of CR proxies; (ii) the relationship between higher scores of CR proxies and better executive performance was progressively mitigated, until disappearing at high levels of L2 proficiency. These results indicate a contribution of bilingualism to CR that spans beyond that of traditional CR proxies.

### Sensitivity Analyses

Both L2 years (β = −2.3; *p* < 0.001) and L2 proficiency (β = −5.723; *p* < 0.001) showed a positive effect on RTs of incongruent trials of the Flanker Task. Moreover, L2 proficiency, but not L2 years, showed a significant interaction with trial type and CR proxies in the modulation analysis (β = 45.124; *p* < 0.001). Thus, the whole-sample sensitivity analyses confirmed our previous results.

## Discussion

The study reported here investigated, for the first time to our knowledge, the effects of bilingualism on executive functioning during senescence operationalizing the bilingual experience as a continuous rather than a categorical variable. This approach, while in line with the general recent trend in bilingualism research (e.g., Hervais-Adelman et al., 2018; DeLuca et al., 2019; Del Maschio et al., 2020; Gallo et al., 2021), was yet to be applied to studies on senior individuals. A continuous assessment of the bilingual experience allows to draw a more detailed, individualized linguistic profile for a life experience that is intrinsically different for each individual, thus contributing to overcoming issues related with inconsistencies in methodological designs and results, which have been plaguing bilingualism research in recent years (Luk and Bialystok, 2013; Mishra, 2015; Surrain and Luk, 2019). Our approach revealed a beneficial effect of increasing L2 proficiency and amount of time passed since acquiring L2 on executive functioning in a sample of bilingual older adults. This result is in line with several previous investigations showing that bilingualism supports the maintenance of optimal executive performance during senescence (e.g., Bialystok et al., 2004; Gold et al., 2013b; Estanga et al., 2017; Del Maschio et al., 2018; Incera and McLennan, 2018). The rationale behind this effect would lie in the extra burden placed on bilinguals’ executive control by the constant necessity to manage crosslinguistic interplay: mechanisms as response selection, interference inhibition, information updating and task-switching have been shown to be constantly active in the bilingual mind and brain during language processing (Abutalebi and Green, 2007; Green and Abutalebi, 2013). This training is thought to lead to ameliorations in bilinguals’ executive network capacity, efficiency and flexibility (for a review see Kroll et al., 2015), namely the action mechanism of CR (Stern, 2009).

Indeed, our findings point to a primary role of bilingualism as a factor supporting CR development, at least in the executive domain. L2 proficiency appeared to modulate the widely known relationship between the most traditional CR proxies, namely level of occupational complexity, maximal educational attainment, social network size and frequency of leisure activities and physical exercise (for a review see Cheng, 2016), and cognition during senescence. For high levels of L2 proficiency, this relationship disappeared, leaving the stage to beneficial effects of bilingualism only, which continued to predict performance level. Our results, indicating a strong contribution of bilingualism to CR development, complement previous findings that dual language use mitigates the relationship between age-related gray (Del Maschio et al., 2018) and white (Gold et al., 2013a) matter deterioration and cognitive decline.

While our results are limited to the executive-function domain, it is key for future research to test whether these effects extend to other cognitive domains and to the cognitive aging trajectory in general. Given the primary role attributed to depletion of executive resources in the cognitive aging process (Davis et al., 2008; Luszcz and Lane, 2008), we hypothesize that bilingualism will prove beneficial for successful aging in general. This hypothesis is supported by evidence that multiple language use supports the maintenance of various non-executive abilities during senescence, such as episodic memory (Wodniecka et al., 2010; Ljungberg et al., 2013), working memory (Bialystok et al., 2014), semantic memory (Arce Rentería et al., 2019), and general intelligence (Bak et al., 2014). Obtaining further evidence is crucial to solidify the presence of bilingualism among widely accepted CR-supporting factors and thus capture the attention of policy makers to reinforce the implementation of bilingual programs. Moreover, although our results highlight a *general* contribution of bilingualism to executive functioning and cognitive reserve, which spans across different language pairs, it is important to direct future efforts toward illuminating the impact of L1-L2 linguistic distance, and other cross-linguistic differences, on the effects presented here. This might further inform policy makers’ attempts to successfully design bilingual interventions and educational policies aimed at mitigating the aging trajectory at various stages of the lifespan.

Indeed, with average life expectancy constantly increasing, age-related cognitive decline is becoming a more and more central issue in our society. Dementia incidence is growing (World Health Organization, 2019), pharmacological solutions to age-related brain pathology are still unsatisfactory (Dyer et al., 2018) and healthcare expenditure dedicated to senior populations is increasingly burdening the public coffers of industrialized countries (Wimo et al., 2017). Thus, the quest for finding non-pharmacological, ecological ways to prevent cognitive aging such as, possibly, bilingualism, must be regarded as an utmost priority by the scientific community.

## Data Availability Statement

The original contributions presented in the study are included in the article/Supplementary Material, further inquiries can be directed to the corresponding author/s.

## Ethics Statement

The studies involving human participants were reviewed and approved by the Ethics Committee at Northumbria University. The patients/participants provided their written informed consent to participate in this study.

## Author Contributions

FG: study conception and design, data analysis, and interpretation. JK: data acquisition. AM: project supervision. All authors: drafting or revising the manuscript for intellectual content, approval of the submitted version.

## Conflict of Interest

The authors declare that the research was conducted in the absence of any commercial or financial relationships that could be construed as a potential conflict of interest.

## Publisher’s Note

All claims expressed in this article are solely those of the authors and do not necessarily represent those of their affiliated organizations, or those of the publisher, the editors and the reviewers. Any product that may be evaluated in this article, or claim that may be made by its manufacturer, is not guaranteed or endorsed by the publisher.

## Footnotes

1
www.prolific.co

2
http://www.cambridgeenglish.org/test-your-english/general-english/
