# Supplementary material for: Add Bilingualism to the Mix: L2 Proficiency Modulates the Effect of Cognitive Reserve Proxies on Executive Performance in Healthy Aging
Source: Front Psychol. 2022 Jan 31;13:780261. doi: 10.3389/fpsyg.2022.780261 (PMC8841471; doi:10.3389/fpsyg.2022.780261)
Supplement: Supplementary file 1 [file Data_Sheet_1.docx]

**Now, you will be asked a few general questions about your demographics**

1. How old are you?

2. What is your sex?

1. Female
2. Male
3. Prefer not to say

3. What is your ethnicity?

4. What is your nationality?

5. Which of the conditions below describes you the best?

1. I only know one language
2. I partially know a second language
3. I know two languages
4. I know more than two languages

6. How old were you when you started learning your second language?

7. How frequently do you use your second language?

1. Daily (more than 2 times/ week)
2. Weekly (more than 2 times/month)
3. Monthly (more than 6 times/year)
4. Quarterly (more than 1 time/ trimester)
5. More rarely
6. I don't know

8. On a scale from 1 to 10, how would you rate your proficiency in your second language?

**Now, you will be asked a few questions about your health**

1. In general, how would you describe your health?

1. poor
2. fair
3. good
4. very good
5. excellent

2. How would you rate your nutritional status?

1. I have no nutritional problems
2. Gravely undernourished/malnourished/obese
3. I don't know

3. Does your general health limit you in moderately strenuous activities, such as moving a table, vacuuming, walking in the forest, or gardening?

1. No, not at all
2. Yes, slightly
3. Yes, severely

4. Does your general health limit you in walking up several flights of stairs?

1. No, not at all
2. Yes, slightly
3. Yes, severely

5. In the last four weeks, have you accomplished less than you wanted, because of your physical health?

1. No
2. Yes

6. In the last four weeks, have you accomplished less than you wanted, because of emotional troubles? (such as anxiety or depression)

1. No
2. Yes

6. Have you been diagnosed with any of the following cardiovascular diseases?

1. Atrial Fibrillation
2. Bradycardias
3. Conduction Diseases
4. Ischemic Heart Disease
5. Cardiac Valve Diseases
6. Heart Failure
7. Stroke
8. Transient Ischemic Attack (TIA) or other Cerebrovascular Disorders
9. Other Cardiovascular Disease
10. No, I have never been diagnosed
11. I don't know

7. If yes, when were you diagnosed with this cardiovascular disease?

8. Have you ever been diagnosed with any of the following neurological disorders?

1. Acute Spinal Cord Injury
2. Alzheimer's Disease
3. Amyotrophic Lateral Sclerosis (ALS)
4. Ataxia
5. Bell's Palsy
6. Brain Tumours
7. Cerebral Aneurysm
8. Epilepsy and Seizures
9. other neurological disorder
10. No, I have never been diagnosed
11. I don't know

9. If yes, when were you diagnosed with this neurological disorder?

10. Have you been diagnosed psychiatric disorders (e.g. Schizophrenia, Bipolar Disorder, Major Depression, Persistent Depressive Disorder, Seasonal Affective Disorder (SAD))?

1. Yes
2. No
3. I don't know

11. If yes, when were you diagnosed with it?

12. Have you been diagnosed with any type of diabetes (e.g. type 1, type 2 and gestational)?

1. Yes
2. No
3. I don't know

13. If yes, when were you diagnosed with it?

**Now you will be asked questions about your social network and leisure activities***.*

1. How often do you personally meet with ... ? (Please, refer to your situation prior to the Covid-19 outbreak)

1. Family
2. Neighbours
3. Friends

Answers to each question:

- 1. Daily (more than 2 times/ week)
  2. Weekly more than 2 times/month
  3. Monthly more than 6 times/year
  4. Quarterly more than 1 time/ trimester
  5. More rarely
  6. Never
  7. I don't know
  8. Not relevant (e.g. has no children or parent no longer alive)

2. How often do you usually have contact by phone, letter / e-mail with … ?(Please, refer to your situation prior to the Covid-19 outbreak)

1. Family
2. Neighbours
3. Friends

Answers to each question:

1. Daily (more than 2 times/ week)
2. Weekly more than 2 times/month
3. Monthly more than 6 times/year
4. Quarterly more than 1 time/ trimester
5. More rarely
6. Never
7. I don't know
8. Not relevant (e.g. has no children or parent no longer alive)

3. Are you happy with your contact with...

1. Family
2. Neighbours
3. Friends

Answers to each question:

- 1. Yes
  2. No
  3. I don't know
  4. Not relevant (e.g. does not have children or parents no longer alive)

4. Can you get help from someone in case of illness or other issue? (e.g. borrow small items, help with repairs, get advice and information)

- 1. Yes, without a doubt
  2. Yes, probably
  3. No, probably not
  4. No, not at all
  5. I don't know

5. How many people do you know well and can talk with them about most things? (e.g. relatives, friends, neighbours and / or co- workers)

- 1. None
  2. 1-2 people
  3. 3 people
  4. 4-6 people
  5. 7-9 people
  6. 10-15 people
  7. 16-30 people
  8. More than 30 people
  9. I don't know

6. Do you feel that you have someone who can give you an adequate personal support to cope with life's stress and problems?

- 1. Yes, without a doubt
  2. Yes, probably
  3. No, probably not
  4. No, not at all
  5. I don't know

7. Are you in a group of friends/acquaintances who have something in common or do something together? (e.g. playing cards, listening to music, travelling etc. )

- 1. Yes
  2. No
  3. Prefer not to say

8. Have you participated in the following activities in the 12 months prior to the outbreak of the

Covid-19 pandemic?

- 1. Cinema/theatre/concert
  2. Sports event
  3. Museum or art exhibition
  4. Visit to restaurant, pub, café
  5. Bingo
  6. Dance
  7. Church/religious meeting
  8. Participated in study circle (group) or course
  9. Participated in non-profit activities
  10. Participated in association work
  11. Travel
  12. Other similar activities

Answers to each question:

- 1. Every week
  2. Every month
  3. More rarely
  4. Never
  5. I don't know

9. Have you participated in the following activities in the 12 months prior to the outbreak of the

Covid-19 pandemic?

- 1. Engaged in gardening
  2. Wander in a forest and picking berries
  3. Hunting or fishing
  4. Knit, weave or sew
  5. Paint, draw etc.
  6. DIY (e.g. repairs at home)
  7. Repair car or machine equipment
  8. Other similar activities

Answers to each question:

1. Every week
2. Every month
3. More rarely
4. Never
5. I don't know

10. Have you participated in the following activities in the 12 months prior to the outbreak of the

Covid-19 pandemic?

- 1. Read a daily newspaper to someone
  2. Read weekly magazines or journals
  3. Read books
  4. Watched TV
  5. Played chess
  6. Played any musical instrument
  7. Listened to music
  8. Use the internet or play computer games
  9. Other similar activities

Answers to each question:

1. Every week
2. Every month
3. More rarely
4. Never
5. I don't know

11. Do you do low-impact exercises? (e.g. walks, short bike rides, light gymnastics, golf)

- The last 12 months (including pandemic period)

- Earlier

Answers to each question:

1. Every week
2. Every month
3. More rarely
4. Never
5. I don't know

12. Do you do more intense exercises – now or earlier?

- The last 12 months (including pandemic period)

- Earlier

Answers to each question:

1. Every week
2. Every month
3. More rarely
4. Never
5. I don't know

**Now, in the last part of this questionnaire, you will be asked questions about your marital status, occupation history, education and upbringing**

13. What is your current marital status?

- 1. Married
  2. Widowed (even after cohabitant)
  3. Unmarried
  4. Divorced
  5. I don't know

14. What is your highest completed level of education?

- 1. Incomplete primary school
  2. Primary school level
  3. High school diploma
  4. University/college with a degree
  5. Postgraduate education
  6. Prefer not to say

15. Are you or were you previously employed?

- 1. Yes
  2. No, housewife/househusband all my life
  3. No, other
  4. Prefer not to say

16. How satisfactory has your professional life been?

- 1. Very satisfactory
  2. Satisfactory
  3. Neither satisfactory nor unsatisfactory
  4. Unsatisfactory
  5. Very unsatisfactory
  6. Prefer not to say

17. What is/was your 'main' occupation?

- Type your 'main job'

18. Did you work after the age of 65?

- 1. Yes
  2. No
  3. Prefer not to say

19. If you answered 'yes' in the previous question, please shortly describe your duties

20.What was your father's main occupation (foster father)?

21. What was your mother’s (foster mother) main occupation during your upbringing?

22. Did you live with both biological parents throughout your upbringing?

- 1. Yes
  2. No
  3. I don't know

23. Did your family have a financially hard time during your upbringing?

- 1. Yes
  2. No
  3. Prefer not to say
